# Hepatic encephalopathy increases the risk for mortality and hospital readmission in decompensated cirrhotic patients: a prospective multicenter study

**DOI:** 10.3389/fmed.2023.1184860

**Published:** 2023-05-25

**Authors:** Oliviero Riggio, Ciro Celsa, Vincenza Calvaruso, Manuela Merli, Paolo Caraceni, Sara Montagnese, Vincenzina Mora, Martina Milana, Giorgio Maria Saracco, Giovanni Raimondo, Antonio Benedetti, Patrizia Burra, Rodolfo Sacco, Marcello Persico, Filippo Schepis, Erica Villa, Antonio Colecchia, Stefano Fagiuoli, Mario Pirisi, Michele Barone, Francesco Azzaroli, Giorgio Soardo, Maurizio Russello, Filomena Morisco, Sara Labanca, Anna Ludovica Fracanzani, Antonello Pietrangelo, Gabriele Di Maria, Silvia Nardelli, Lorenzo Ridola, Antonio Gasbarrini, Calogero Cammà

**Affiliations:** ^1^Department of Translational and Precision Medicine, Sapienza University of Rome, Rome, Italy; ^2^Department of Gastroenterology, “Santa Maria Goretti” Hospital, “Sapienza” Polo Pontino, Latina, Italy; ^3^Section of Gastroenterology and Hepatology, Department of Health Promotion, Mother and Child Care, Internal Medicine and Medical Specialties, PROMISE, University of Palermo, Palermo, Italy; ^4^Department of Surgical, Oncological and Oral Sciences (Di.Chir.On.S.), University of Palermo, Palermo, Italy; ^5^Istituto di Ricovero e Cura a Carattere Scientifico (IRCCS) Azienda Ospedaliera-Universitaria di Bologna, Bologna, Italy; ^6^Department of Medical and Surgical Sciences, Center for Biomedical Applied Research, Alma Mater Studiorum University of Bologna, Bologna, Italy; ^7^Department of Medicine, University of Padova, Padua, Italy; ^8^Medicina Interna e Gastroenterologia, Università Cattolica del Sacro Cuore, Fondazione Policlinico Universitario Gemelli Istituto di Ricovero e Cura a Carattere Scientifico (IRCCS), Rome, Italy; ^9^Hepatology and Liver Transplant Unit, University of Tor Vergata, Rome, Italy; ^10^Division of Gastroenterology, Department of Medical Sciences, University of Turin, Turin, Italy; ^11^Division of Medicine and Hepatology, University Hospital of Messina, Messina, Italy; ^12^Department of Gastroenterology and Hepatology, Università Politecnica delle Marche, Ancona, Italy; ^13^Gastroenterology/Multivisceral Transplant Unit, Department of Surgery, Oncology and Gastroenterology, Padua University Hospital, Padua, Italy; ^14^Gastroenterology Unit, Department of Surgical and Medical Sciences, University of Foggia, Foggia, Italy; ^15^Internal Medicine and Hepatology Unit, Department of Medicine, Surgery and Dentistry, “Scuola Medica Salernitana”, University of Salerno, Salerno, Italy; ^16^Gastroenterology Unit, Department of Medical Specialities, University Hospital of Modena, University of Modena and Reggio Emilia, Modena, Italy; ^17^Unit of Gastroenterology, Borgo Trento University Hospital, Verona, Italy; ^18^Gastroentyerology, University of Milan Bicocca, Milan, Italy; ^19^Gastroenterology, Hepatology and Transplantation Unit, Papa Giovanni XXIII Hospital, Bergamo, Italy; ^20^Department of Translational Medicine, Università del Piemonte Orientale UPO, Novara, Italy; ^21^Internal Medicine Unit, Azienda Ospedaliera Universitaria (AOU) Maggiore della Carità Hospital, Novara, Italy; ^22^Section of Gastroenterology, Department of Emergency and Organ Transplantation, University “Aldo Moro” of Bari, Bari, Italy; ^23^Gastroenterology Unit, Istituto di Ricovero e Cura a Carattere Scientifico (IRCCS) Azienda Ospedaliero-Universitaria di Bologna, Department of Surgical and Medical Sciences, Alma Mater Studiorum University of Bologna, Bologna, Italy; ^24^Clinic of Internal Medicine-Liver Unit, Department of Medical Area (DAME), University of Udine, Udine, Italy; ^25^Italian Liver Foundation, Area Science Park, Trieste, Italy; ^26^Liver Unit, Azienda di Rilievo Nazionale ed Alta Specializzazione (ARNAS) Garibaldi-Nesima, Catania, Italy; ^27^Gastroenterology and Hepatology Unit, Department of Clinical Medicine and Surgery, University of Naples “Federico II”, Naples, Italy; ^28^Gastroenterology Unit, Department of Internal Medicine, University of Genoa, Genoa, Italy; ^29^Department of Pathophysiology and Transplantation, Università degli Studi di Milano, Milan, Italy; ^30^General Medicine and Metabolic Diseases, Fondazione IRCCS Ca' Granda Ospedale Maggiore Policlinico, Milan, Italy; ^31^Department of Internal and Emergency Medicine, University Hospital of Modena, Modena, Italy

**Keywords:** hepatic encephalopathy, decompensated cirrhosis, orthotopic liver transplant, hospital readmission, mortality

## Abstract

**Introduction:**

Hepatic encephalopathy (HE) affects the survival and quality of life of patients with cirrhosis. However, longitudinal data on the clinical course after hospitalization for HE are lacking. The aim was to estimate mortality and risk for hospital readmission of cirrhotic patients hospitalized for HE.

**Methods:**

We prospectively enrolled 112 consecutive cirrhotic patients hospitalized for HE (HE group) at 25 Italian referral centers. A cohort of 256 patients hospitalized for decompensated cirrhosis without HE served as controls (no HE group). After hospitalization for HE, patients were followed-up for 12 months until death or liver transplant (LT).

**Results:**

During follow-up, 34 patients (30.4%) died and 15 patients (13.4%) underwent LT in the HE group, while 60 patients (23.4%) died and 50 patients (19.5%) underwent LT in the no HE group. In the whole cohort, age (HR 1.03, 95% CI 1.01–1.06), HE (HR 1.67, 95% CI 1.08–2.56), ascites (HR 2.56, 95% CI 1.55–4.23), and sodium levels (HR 0.94, 95% CI 0.90–0.99) were significant risk factors for mortality. In the HE group, ascites (HR 5.07, 95% CI 1.39–18.49) and BMI (HR 0.86, 95% CI 0.75–0.98) were risk factors for mortality, and HE recurrence was the first cause of hospital readmission.

**Conclusion:**

In patients hospitalized for decompensated cirrhosis, HE is an independent risk factor for mortality and the most common cause of hospital readmission compared with other decompensation events. Patients hospitalized for HE should be evaluated as candidates for LT.

## Introduction

Hepatic encephalopathy (HE) is a well-known relevant complication of cirrhosis, and it includes a wide spectrum of neuropsychiatric abnormalities ranging from subclinical alterations to coma (1). Although the prevalence and cumulative incidence of HE are difficult to assess, it has been estimated that 35–45% of patients with cirrhosis could experience overt HE, with an incidence of 11.6 per 100 person-years, rising to 40% by 5 years (2, 3). HE has a significant impact on the risk of hospitalization, accidental trauma, and survival, with a mortality rate of ~60% at 1 year (4–6). HE has a significant impact not only on survival but also on the quality of life of patients and their caregivers, and it is associated with a significant financial burden for healthcare systems, increasing both direct and indirect costs (7–9). Current management strategies include the identification, prevention, and treatment of precipitating factors, the use of lactulose and rifaximin, both as prophylaxis and treatment measures, and finally liver transplantation in patients with end-stage liver disease and recurrent or persistent HE not responding to other treatments (1).

Despite its clinical, social, and economic relevance, data on the impact of HE on the clinical course of patients with decompensated cirrhosis still remain scanty. Particularly, data on the risk for HE recurrence come mainly from small retrospective studies, not specifically designed for this purpose and without a clearly defined inception point. Moreover, although it is well known that a substantial proportion of episodes of HE is related to the presence of precipitating factors, such as constipation, infections, gastrointestinal bleeding, and diuretic overuse, the relative weight of these precipitating events, as well as their prevalence, remain poorly known (10).

Therefore, prospective data collected from a well-defined inception point (i.e., hospitalization for HE with or without a history of HE) evaluating the impact of HE on mortality, the access to orthotopic liver transplant (OLT), and the risk of hospital readmission are lacking. Most of the previously published studies are affected by their retrospective design, the administrative nature of data, or the inclusion of patients with heterogeneous characteristics, in terms of severity of liver disease and characteristics of HE. Moreover, an accurate estimate of the impact of HE recurrence as a decompensation event leading to further hospitalization is relevant, in order to assess the cost-effectiveness of secondary prophylaxis strategies.

This multicenter prospective study, including a cohort of patients with decompensated cirrhosis hospitalized for HE, aimed to estimate the mortality, the rate of access to OLT, and the risk for hospital readmission in patients with HE compared with patients with other decompensation events of cirrhosis.

## Materials and methods

### Study design and patients

This observational, prospective, multicenter, and investigator-driven cohort study enrolled all consecutive patients with cirrhosis hospitalized for grade ≥ 2 HE [according to the West Haven criteria (11)] at 25 Italian centers between January 2019 and November 2020 (HE group). Another cohort of patients hospitalized for decompensated cirrhosis (i.e., ascites, portal hypertensive bleeding, and jaundice), without HE at the time of hospitalization and during the previous 12 months, observed at the same centers during the same period served as the control group (no HE group).

Exclusion criteria were as follows: (a) age lower than or equal to 17 years; (b) age higher than or equal to 79 years; (c) non-cirrhotic portal hypertension; (d) acute liver failure; (e) psychiatric or neurodegenerative diseases; (f) alteration of mental status not related to HE; and (g) participation to clinical trials evaluating therapeutic interventions for HE.

Participating centers were chosen on the basis of their recognized expertise as high-volume tertiary referral centers for the management of patients with decompensated cirrhosis.

Patient data were recorded by treating clinicians from each participating center at the inception point (i.e., the index hospitalization for HE or other complications of cirrhosis in patients with or without history of liver decompensation). Data were collected in a dedicated and anonymized electronic case report form (CRF), shared by all the participating centers. Demographics, educational level, working activities, body mass index (BMI), etiology of cirrhosis, biochemistry (liver function tests, serum creatinine, sodium, and venous blood ammonia), presence and severity of ascites and HE, Child-Pugh class, model for end-stage liver disease (MELD) and MELD-Na scores, presence and severity of portal hypertension (esophageal and/or gastric varices, portal vein and spleen diameters, portosystemic shunts), portal vein thrombosis, hepatocellular carcinoma (HCC), and previous pharmacological treatments were recorded.

The diagnosis of HE was performed according to the guidelines of American Association for the Study of Liver Diseases and the European Association for the Study of the Liver (12) and by using a flow-chart for differential diagnosis between HE and other causes of neuropsychiatrist alterations, not related to HE (see Supplementary material).

The presence of esophageal and/or gastric varices was evaluated by upper endoscopy. Portal vein and spleen diameters were evaluated by abdominal ultrasound. Porto-systemic shunts and portal vein thrombosis were evaluated by abdominal contrast-enhanced computed tomography (CT), when available. The diagnosis of HCC was performed according to the European guidelines (13).

In the HE group, data on the previous history of HE, the clinical course of previous HE [episodic, recurrent, or persistent, defined according to the International Guidelines (12)], previous hospitalizations, grading of HE according to the West Haven criteria (11), animal name testing (ANT), Glasgow Coma Scale (GCS), duration of HE episode, number and types of precipitating factors, treatments received, and secondary prophylaxis were recorded. The definition of precipitating factors is presented in Supplementary material.

The study was approved by the Institutional Review Board (IRB) of the Fondazione Agostino Gemelli Hospital. Informed consent to participate in the study was acquired according to the following modalities: (a) patients with preserved state of consciousness gave their written consent to participate in the study at the time of enrollment; (b) patients with HE at the time of enrollment who were unable to express consent have been registered as potential candidates without extracting data from the medical record, and a retroactive consent has been collected from the patient at the time of the recovery of cognitive conditions, or from his legal representative in case of persistence of inability to provide consent; (c) for patients who died following the HE episode without expressing consent, an authorization from the ethics committee for the exemption from informed consent for the use of data, were obtained.

### Follow-up and outcomes

After the index hospitalization, all patients were followed-up for 12 months or until death or orthotopic liver transplant (OLT). Follow-up protocol included telephone follow-up evaluations performed every month by treating clinicians and medical reviews including physical examination (including assessment of HE and its grading) and biochemistry evaluation every 3 months. During follow-up, causes and dates of hospital readmissions were recorded.

The primary outcome was mortality, with OLT considered as a competing event.

The secondary outcome was hospital readmission for further decompensation events both in the HE and no HE groups. Further decompensation events were defined as HE, ascites, infections, and portal hypertensive bleeding. Infections were defined as spontaneous bacterial peritonitis, urinary tract infections, pneumonia, and bacteremia.

### Statistical analysis

Data for continuous variables are expressed as mean (standard deviation) or median (interquartile range). Data for categorical variables are expressed as frequency (percentage).

Mortality and OLT were evaluated by competing risks survival analysis and represented by cumulative incidence function (CIF) (14). Cox cause-specific model was fitted in order to estimate the effect of covariates on mortality. Covariates used for multivariable analyses were chosen based on their significance in the univariate analysis (*p* < 0.10). Covariates in the final model with a *p*-value of < 0.05 were considered statistically significant. The results are presented as adjusted hazard ratios (HR) and their 95% confidence intervals (CI). Composite covariates (i.e., Child–Pugh class, MELD) were not included in the multivariable model to avoid collinearity with the individual score items. To take into account the between-center heterogeneity, a multivariable model including the center as a covariate was fitted, with centers categorized according to the number of enrolled patients (10 or less patients, between 11 and 20 patients, and more than 20 patients).

Risk factors for mortality identified by competing risks multivariable analysis were used to generate a prediction rule. The predicted probability of dying was computed for a hypothetical patient, identified by a combination of prognostic factors.

The probability of hospital readmission for further decompensating events was evaluated by competing risks analysis, represented by CIF. In this analysis, the event of interest was the first decompensation event that occurred during the follow-up, leading to hospital readmission.

Details on sample size calculation are presented in Supplementary material.

All analyses were performed in R core Team (version 4.0.3).

## Results

### Baseline

The study flowchart is shown in Supplementary Figure S1. The final study population included 368 patients (HE group: 112 patients; no HE group: 256 patients), and their baseline characteristics are shown in Table 1. The mean age was 60.4 ± 10.4, and 72% of patients were male. Alcohol-related liver disease was the most common etiology (38%), followed by HCV infection (15%). Most of the patients had Child–Pugh classes B and C (43 and 39%, respectively), and the mean MELD score was 17.8 ± 7.6. Esophageal varices were present in 72% of patients. CT scan was available in 213 patients, and porto-systemic shunts and portal vein thrombosis were found, respectively, in 42% and 20% of patients who underwent CT scan.

**Table 1:** Baseline characteristics of 368 cirrhotic patients admitted to hospital for hepatic encephalopathy (HE) (HE group) or for other decompensating events (no HE group).

**Variable**	**Overall cohort (*N* = 368)**	**HE group (*n* = 112)**	**No HE group (*n* = 256)**	***p*-value**
Age (years)	60.4 ± 10.4	62.3 ± 8.3	59.6 ± 11.1	0.03
Male sex (%)	266 (72.3)	91 (81.2)	175 (68.4)	0.01
BMI (kg/m^2^)	26.9 ± 5.6	25.7 ± 4.3	27.4 ± 5.9	0.02
**Education (%)**				0.75
Primary school	111 (30.2)	38 (33.9)	73 (28.5)	
Middle school	137 (37.2)	37 (33.0)	100 (39.1)	
High school	93 (25.3)	28 (25.0)	65 (25.4)	
University	18 (4.9)	6 (5.4)	12 (4.7)	
Other	9 (2.4)	3 (2.7)	6 (2.3)	
**Job (%)**				0.31
Clerk	12 (3.3)	3 (2.7)	9 (3.5)	
Freelance	18 (4.9)	4 (3.6)	14 (5.5)	
Manager	4 (1.1)	1 (0.9)	3 (1.2)	
Workman	93 (25.3)	25 (22.3)	68 (26.6)	
Retired	133 (36.1)	50 (44.6)	83 (32.4)	
Unemployed	108 (29.3)	29 (25.9)	79 (30.9)	
**Etiology of liver disease (%)**				0.34
HCV	56 (15.2)	18 (16.1)	38 (14.8)	
HBV	27 (7.3)	5 (4.5)	22 (8.6)	
Alcohol	140 (38.0)	42 (37.5)	98 (38.3)	
Viral + alcohol	51 (13.9)	14 (12.5)	37 (14.5)	
Metabolic	44 (11.9)	11 (9.8)	33 (12.9)	
Autoimmune hepatitis	11 (3.00)	6 (5.4)	5 (1.9)	
Biliary diseases	13 (3.5)	5 (4.5)	8 (3.1)	
Others	26 (7.1)	11 (9.8)	15 (5.9)	
**Complications and severity of cirrhosis**				
Ascites absent	117 (31.8)	36 (32.1)	81 (31.6)	
Grade 1–2 ascites	177 (48.1)	62 (55.4)	115 (44.9)	0.09
Grade 3–4 ascites	74 (20.1)	14 (12.5)	60 (23.4)	
Portal thrombosis^**^	44 (20.6)	16 (27.1)	28 (18.2)	0.15
Presence of TIPS	16 (4.3)	11 (9.8)	5 (2.0)	< 0.001
Esophageal varices absent	104 (28.2)	30 (26.7)	74 (28.9)	
F1 esophageal varices	117 (31.8)	42 (37.5)	75 (29.3)	0.87
F2 Esophageal varices	119 (32.3)	31 (27.7)	88 (34.4)	
F3 Esophageal varices	28 (7.6)	9 (8.0)	19 (7.4)	
GOV	39 (10.6)	12 (10.7)	27 (10.5)	
IGV	8 (2.2)	2 (1.7)	6 (2.3)	
Hepatocellular carcinoma^**^	72 (33.8)	12 (20.3)	60 (39.0)	0.01
Child-Pugh score	8.7 ± 2.1	9.9 ± 1.8	8.2 ± 2.0	< 0.001
Child-Pugh class A	67 (18.2)	5 (4.5)	62 (24.2)	
Child-Pugh class B	158 (42.9)	40 (35.7)	118 (46.1)	< 0.001
Child-Pugh class C	143 (38.9)	68 (60.7)	75 (29.3)	
MELD score	17.8 ± 7.6	20.2 ± 7.4	16.7 ± 7.5	< 0.001
MELD-Na score	19.4 ± 7.8	21.8 ± 7.4	18.3 ± 7.7	< 0.001
**Biochemistry**				
Hemoglobin (g/dL)	10.3 ± 1.9	10.0 ± 2.0	10.4 ± 1.9	0.05
Haematocrit (%)	31 ± 5.7	29.5 ± 5.6	31.6 ± 5.7	0.002
WBC (mmc)	5,660 ± 3,798	5,978 ± 3,234	5,521 ± 4,017	0.29
PLT (10^9^/L)	93.6 ± 63.7	85.6 ± 58.1	97.0 ± 65.8	0.12
ALT (U/mL)	43 ± 51	40 ± 31	44 ± 57	0.59
AST (U/mL)	64 ± 75	60 ± 68	66 ± 78	0.5
GGT (U/mL)	109 ± 138	80 ± 76	120 ± 155	0.01
Total bilirubin (mg/dL)	4.6 ± 6.4	5.3 ± 6.3	4.2 ± 6.4	0.14
Creatinine (mg/dL)	1.05 ± 0.7	1.14 ± 0.71	1.01 ± 0.69	0.11
Albumin (g/dL)	3.1 ± 0.6	2.9 ± 0.5	3.1 ± 0.6	0.01
INR	1.6 ± 0.4	1.6 ± 0.5	1.5 ± 0.4	0.1
Sodium (mEq/L)	137 ± 4	136 ± 5	137 ± 4	0.29
Venous blood ammonia (μmol/L)	109 ± 69	130 ± 76	92 ± 59	< 0.001
**Non-invasive indicators of portal hypertension**				
Portal vein diameter (mm)^*^	13.4 ± 7.8	13.3 ± 3.0	13.8 ± 2.7	0.3
Spleen diameter (cm)^**^	15.5 ± 3.1	15.7 ± 3.3	15.4 ± 3.0	0.59
Porto-systemic shunts^**^	90 (42.2)	33 (55.9)	57 (37.0)	0.02
**Ongoing pharmacological treatments (%)**				
Propranolol	120 (32.6)	31 (27.7)	89 (34.8)	0.41
Carvedilol	47 (12.8)	17 (15.2)	30 (11.7)	0.64
Furosemide	250 (67.9)	81 (72.3)	169 (66.0)	0.45
Spironolactone	31 (8.4)	11 (9.8)	20 (7.8)	0.8
Canrenone	85 (23.1)	25 (23.2)	60 (23.4)	0.96
Disaccharides	218 (59.2)	91 (81.3)	127 (49.6)	< 0.001
Rifaximin	151 (41.0)	66 (58.9)	51 (19.9)	< 0.001
PPI	224 (60.9)	75 (67.0)	149 (58.2)	0.26
Albumin	79 (21.5)	27 (24.1)	52 (20.3)	0.7
Branched-chain aminoacids	22 (6.0)	11 (9.8)	11 (4.3)	0.12

The results are presented as mean ± standard deviation for continuous variables or absolute number (percentage) for categorical variables.

HE, hepatic encephalopathy; HCV, hepatitis C virus; HBV, hepatitis B virus; BMI, body mass index; WBC, white blood cell; PLT, platelet; ALT, alanine aminotransferases; AST, aspartate aminotransferases; GGT, gamma-glutamil transferase; INR, international normalized ratio; MELD, model for end-stage liver disease; GOV, gastro-esophageal varices; IGV, isolated gastric varices; TIPS, transjugular intrahepatic porto-systemic shunt; PPI, proton pump inhibitors.

^*^Assessed by ultrasonography.

^**^Assessed by computed tomography (available in 213 patients: 59 patients with HE and 154 patients without HE).

Compared with the no HE group, patients in the HE group were significantly older, more frequently male, and they had a significantly poorer nutritional status (lower BMI and albumin levels). The HE group displayed signs of more advanced liver disease, as shown by a significantly higher prevalence of Child–Pugh class C and higher MELD and MELD-Na scores. Venous blood ammonia levels were significantly higher in the HE group compared with the no HE group. Porto-systemic shunts and TIPS were significantly more frequent in the HE group.

The main clinical features and treatments of the HE group are shown in Table 2. Most of the patients (59%) had a previous history of HE, which was episodic in 13%, recurrent in 42%, and persistent in 4% of patients. Conversely, 41% of patients were at the first HE episode. At least one precipitating factor was identified in 89 patients (79.5%). The most common precipitating factors were constipation (29.5%), infections (20.5%), and diuretics overuse (17%). Most of the patients (65%) had grade 2 HE, median ANT was 6, and median GCS was 14. The most common treatments used were lactulose (oral and enema in 72% and 53% of patients, respectively) and rifaximin in 67% of patients. The median duration of the HE episode was 1.8 days. During hospitalization, HE resolved in 92.8% of patients and recurred in 4.5% of patients, while 2.7% of patients died. Most of the patients (81%) received secondary prophylaxis, including rifaximin in 73% and non-absorbable disaccharides in 61% of patients.

**Table 2:** Clinical features and treatments in 112 cirrhotic patients hospitalized for hepatic encephalopathy (HE group).

	**Patients with HE (*n* = 112)**
First HE episode (*n*, %)	46 (41.1)
Previous history of HE (*n*, %)	66 (58.9)
Episodic HE	15 (13.4)
Recurrent HE	47 (42.0)
Persistent HE	4 (3.6)
Number of HE episodes during last 6 months (median, range)	2 (1-6)
**Precipitating events**	
No precipitating events (*n*, %)	23 (20.5)
Precipitating events (*n*, %)	89 (79.5)
Constipation	33 (29.5)
Infection	23 (20.5)
Diuretics/dehydration	19 (17.0)
Gastrointestinal bleeding	14 (12.5)
**HE staging**	
Animal name testing (ANT) (median, range)	6 (0–19)
ANT < 10 (*n*, %)	85 (75.9)
Glasgow Coma Scale (GCS) (median, range)	14 (3–15)
HE grade (*n*, %)	
Grade 2	73 (65.2)
Grade 3	36 (32.1)
Grade 4	3 (2.7)
**Treatments for HE episode (** ***n*** **, %)**	
Lactulose	81 (72.3)
Rifaximin	75 (67.0)
Lactulose enema	60 (53.6)
Albumin	46 (41.1)
Branched-chain aminoacids	44 (39.3)
Systemic antibiotics	34 (30.4)
Lactitol	11 (9.8)
Fasting	8 (7.1)
Nutritional support	4 (3.6)
Low protein diet	3 (2.7)
**Treatments for precipitating event (** ***n*** **, %)**	
**Treatments for infection**	
Piperacillin/tazobactam	8 (7.1)
Ceftriaxone	7 (6.3)
Ciprofloxacin	4 (3.6)
Cefotaxime	2 (1.8)
Meropenem	2 (1.8)
Fosfomycin	2 (1.8)
Cefepime	1 (0.9)
Ceftobiprole	1 (0.9)
Tigecycline	1 (0.9)
Not specified antibiotics	4 (3.6)
**Treatments for electrolytes alteration**	
Sodium or potassium replacement	5 (4.5)
**Treatments for gastrointestinal bleeding**	
Blood transfusion	4 (3.6)
Vasoactive treatment (somatostatin or terlipressin)	4 (3.6)
Endoscopic variceal ligation	3 (2.7)
Endoscopic treatment of peptic ulcer	3 (2.7)
Endoscopic treatment of GAVE	1 (0.9)
**Treatments for diuretic overuse**	
Diuretic discontinuation	8 (7.1)
Diuretic dose reduction	7 (6.3)
**Treatments for constipation**	
Lactulose enema	36 (32.1)
Oral lactulose	23 (20.5)
**Treatments for dehydration**	
Intravenous fluid infusion	17 (15.2)
**Outcomes of HE episode during hospitalization (** ***n*** **, %)**	
Resolution	104 (92.8)
Recurrence of HE	5 (4.5)
Death	3 (2.7)
**Secondary prophylaxis after HE episode (** ***n*** **, %)**	88 (80.7)
Rifaximin	80 (73.3)
Non-adsorbable disaccharides	67 (61.5)
Low-protein diet	11 (10.0)
Vegetal protein diet	8 (7.3)
Branched-chain Aminoacids	2 (1.8)

HE, hepatic encephalopathy; GAVE, Gastric Antral Vascular Ectasia.

### Outcomes

#### Mortality

Outcomes during follow-up are shown in Table 3. In the whole cohort, 94 patients (25.5%) died and 65 patients (17.7%) underwent OLT. Cumulative probabilities of death and OLT are shown in Supplementary Figure S2. Six- and twelve-month probabilities of death were 22.4% and 29.1%, respectively, and 6- and 12-month probabilities of OLT were 14.9% and 21.2%, respectively.

**Table 3:** Outcomes of 368 hospitalized patients with decompensated cirrhosis according to the presence or absence of hepatic encephalopathy (HE).

	**Overall cohort (*N* = 368)**	**HE group (*n* = 112)**	**No HE group (*n* = 256)**
Death (*n*, %)	94 (25.5)	34 (30.4)	60 (23.4)
Liver transplant (*n*, %)	65 (17.7)	15 (13.4)	50 (19.5)
At least one hospital readmission (*n*, %)	107 (29.1)	39 (34.8)	68 (26.6)
**Number of hospital readmissions**			
1	66 (17.9)	23 (20.5)	43 (16.8)
2	25 (6.8)	9 (8.0)	16 (6.3)
≥3	16 (4.3)	7 (6.3)	9 (3.5)
**Reasons of hospital readmissions (** ***n*** **, %)**			
HE	51 (13.9)	24 (21.4)	27 (10.5)
Ascites	42 (11.4)	15 (13.4)	27 (10.5)
Infections	17 (4.6)	4 (3.6)	13 (5.1)
Portal hypertensive bleeding	18 (4.9)	3 (2.7)	15 (5.9)

HE, hepatic encephalopathy.

**Figure 1:**
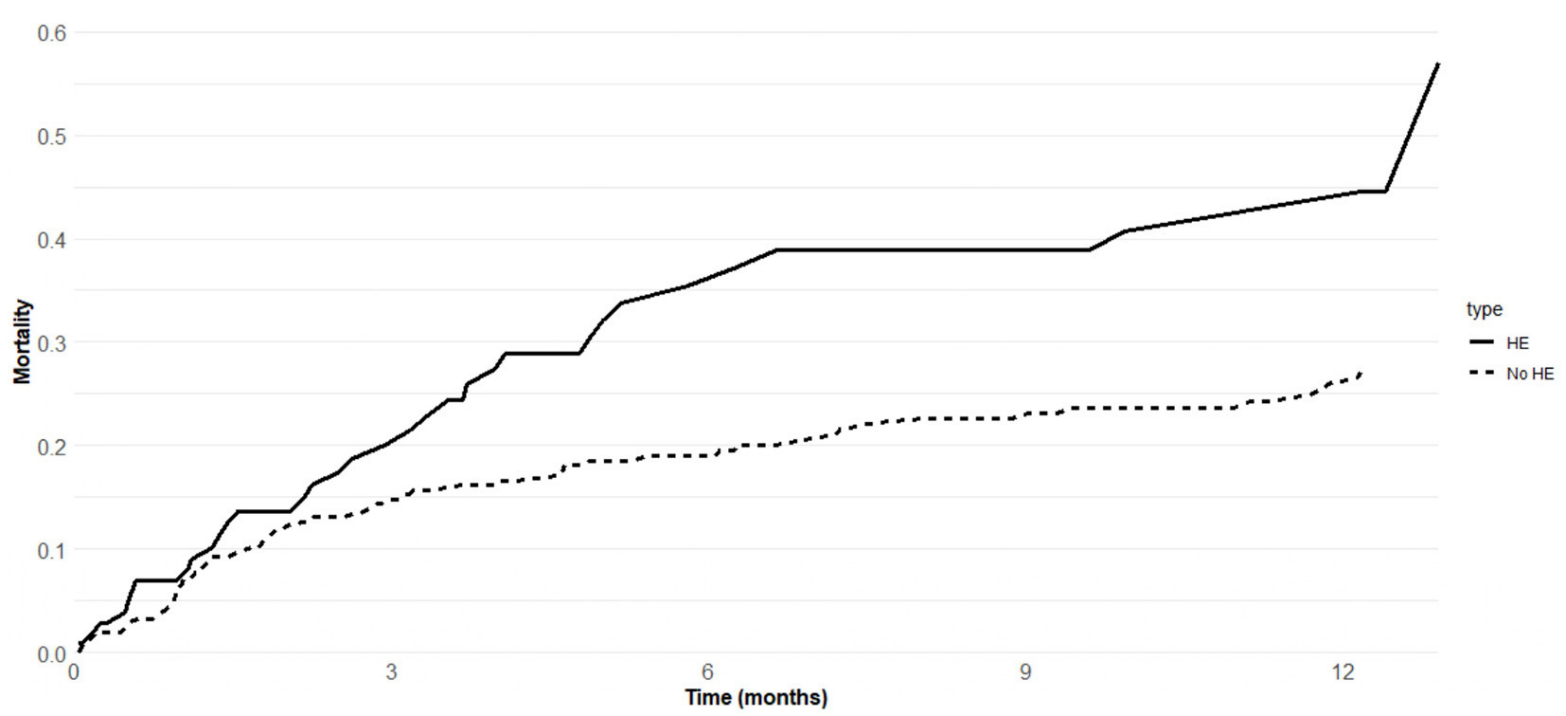
Mortality of 368 cirrhotic patients admitted to hospital for hepatic encephalopathy (HE group) or other decompensating events (no HE group).

In the HE group, 34 patients (30.4%) died and 15 patients (13.4%) underwent OLT. In the no HE group, 60 patients (23.4%) died and 50 patients (19.5%) underwent OLT. Figure 1 showed probabilities of death in the HE and no HE groups. Six- and twelve-month probabilities of death were higher in the HE group [34.3% (95% CI 30.4–37.5%) and 40.8% (95% CI 38.4–43.2%), respectively] than in the no HE group [18.7% (95% CI 14.8–21.3%) and 26.3% (95% CI 22.4–28.5%), respectively]. Six- and twelve-month probabilities of OLT were 10.0% (95% CI 8.8–13.4%) and 16.3% (95% CI 13.8–17.9%) in the HE group, respectively, and 16.5% (95% CI 12.4–17.8%) and 22.9% (95% CI 19.8–24.8%) in the no HE group (Supplementary Figure S3).

Univariate analysis of risk factors for death and OLT is presented in Supplementary Table S1. In the multivariable model, four variables were independently associated with mortality as follows: age (HR 1.03, 95% CI 1.01–1.06, *p* = 0.018), HE (HR 1.67, 95% CI 1.08–2.56, *p* = 0.020), sodium (HR 0.94, 95% CI 0.90–0.99, *p* = 0.013), and presence of ascites (HR 2.56, 95% CI 1.55–4.23, *p* = 0.012) (Table 4). Similar results were obtained when HE status was codified as first or episodic HE (i.e., patients at first HE episode or with a history of episodic HE. HR 1.82, 95% CI 1.02–3.27, *p* = 0.043) and recurrent or persistent HE (HR 1.80, 95% CI 1.05–3.10, *p* = 0.034), with the no HE group as reference (Table 4). Moreover, age (HR 1.03, 95% CI 1.01–1.05, *p* = 0.031), HE (HR 1.62, 95% CI 1.01–2.58, *p* = 0.043), ascites (HR 2.75, 95% CI 1.59–4.76, *p* < 0.001), and sodium (HR 0.95, 95% CI 0.91–0.99, *p* = 0.019) were confirmed as independent predictors of mortality when covariates significantly different between the HE and no HE groups, and potentially affecting survival was included in the multivariate model [i.e., male sex (HR 1.26, 95% CI 0.75–2.09, *p* = 0.379), BMI (HR 0.99, 95% CI 0.95–1.04, *p* = 0.784), HCC (HR 1.37, 95% CI 0.78–2.39, *p* = 0.273), TIPS (HR 0.67, 95% CI 0.18–2.54, *p* = 0.553), hemoglobin (HR 1.02, 95% CI 0.91–1.14, *p* = 0.723), GGT (HR 1, 95% CI 0.99–1.01, *p* = 0.058), albumin (HR 1.02, 95% CI 0.69–1.49, *p* = 0.936), and porto-systemic shunts (HR 0.93, 95% CI 0.57–1.52, *p* = 0.765)] (Supplementary Table S2).

**Table 4:** Risk factors for mortality and liver transplant in the whole cohort of cirrhotic patients (*n* = 368) hospitalized for decompensating events and in 112 patients hospitalized for hepatic encephalopathy (HE group) by multivariable competing risks analysis.

**Whole cohort** ^*^						
	**Death**			**Liver transplant**		
	**HR**	**95% CI**	***p*** **-value**	**HR**	**95% CI**	***p*** **-value**
Age (years)	1.03	1.01–1.05	0.025	0.96	0.94–0.99	0.002
HE (present vs. absent)	1.69	1.09–2.62	0.018	1.27	0.70–2.30	0.437
Sodium (mEq/L)	0.94	0.90–0.98	0.009	0.97	0.91–1.03	0.261
Ascites (present vs. absent)	2.50	1.49–4.18	< 0.001	0.87	0.51–1.49	0.613
**HE cohort** ^**^						
	**Death**			**Liver transplant**		
	**HR**	**95% CI**	***p*** **-value**	**HR**	**95% CI**	***p*** **-value**
BMI (kg/m^2^)	0.86	(0.75–0.98)	0.027	1	(0.85–1.17)	0.960
Ascites (present vs. absent)	5.07	(1.39–18.49)	0.014	0.39	(0.09–1.66)	0.203

In the whole cohort, similar results were obtained when HE status was codified as first or episodic HE (i.e., patients at first HE episode or with history of episodic HE. HR 1.82, 95% CI 1.02–3.27, *p* = 0.043) and recurrent or persistent HE (HR 1.80, 95% CI 1.05–3.10, *p* = 0.034), with No HE group as reference.

HE, hepatic encephalopathy; BMI, body mass index; HR, hazard ratio; CI, confidence interval.

^*^The results are adjusted for center effect.

^**^The results are adjusted for infections as precipitating events and for the presence of hepatocellular carcinoma.

Supplementary Figure S4 shows the predicted probabilities of death in four different patient profiles according to the presence of HE and/or ascites. In patients without HE and ascites, 6- and 12-month probabilities of death were 11.3% and 15.5%, respectively; in patients with HE and without ascites, 6- and 12-month probabilities of death were 18.8% and 25.4%, respectively; in patients without HE and with ascites, 6- and 12-month probabilities of death were 24.6% and 32.6%, respectively; in patients with HE and ascites, 6- and 12-month probabilities of death were 38.8% and 49.2%, respectively.

In the HE group, BMI (HR 0.86, 95% CI 0.75–0.98, *p* = 0.027) and ascites (HR 5.07, 95% CI 1.39–18.49, *p* = 0.014) were independently associated with mortality (Table 4).

#### Hospital readmissions

**Figure 2:**
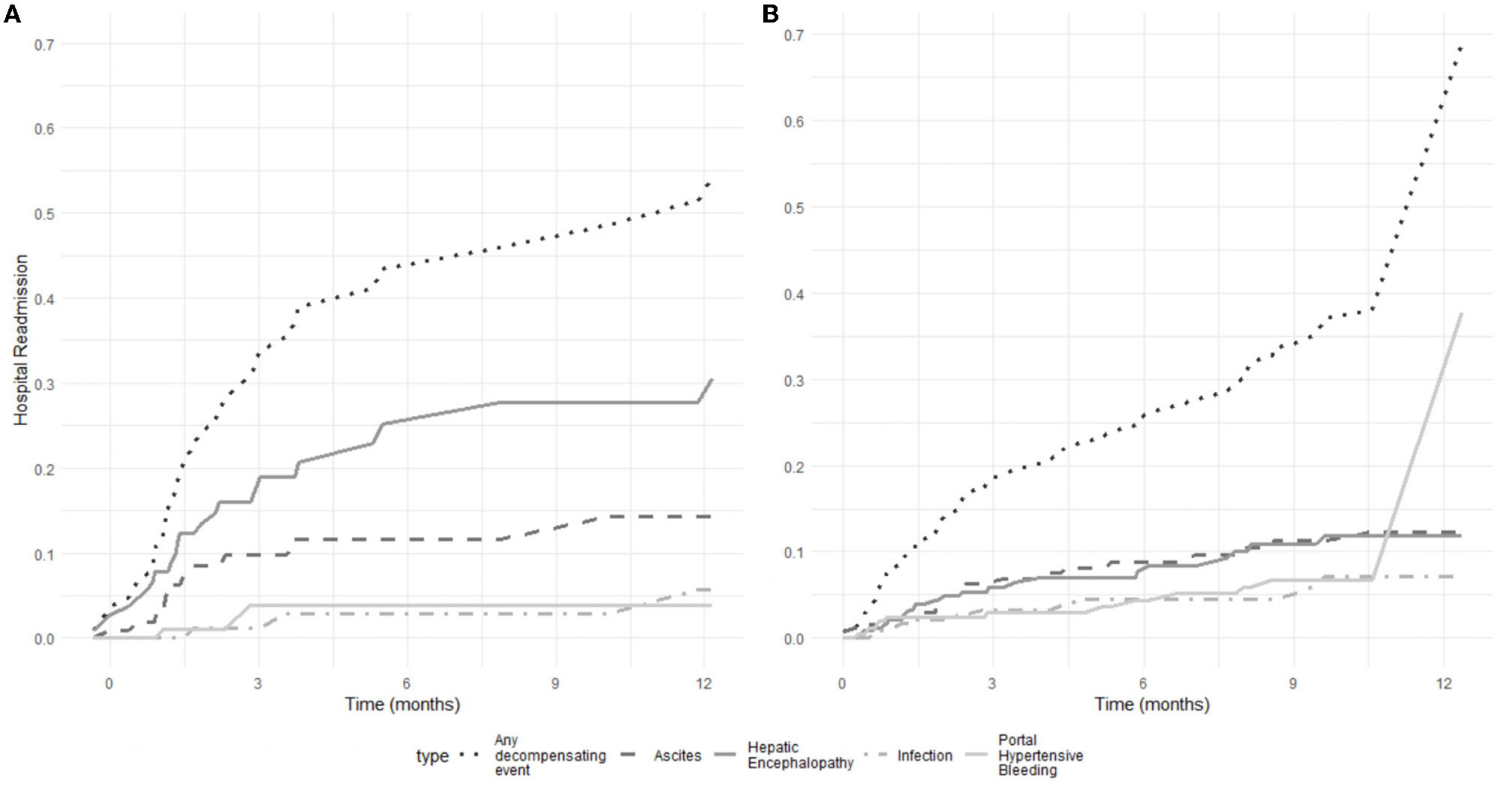
Probability of hospital readmission for further decompensating events (hepatic encephalopathy, ascites, infections, and portal hypertensive bleeding) in 112 patients with cirrhosis hospitalized for hepatic encephalopathy **(A)** and 256 patients hospitalized for decompensated cirrhosis without hepatic encephalopathy **(B)**.

During follow-up, in the HE group, 39 patients (34.8%) had at least one hospital readmission, while 73 patients (65.2%) had no hospital readmission. The reasons for hospital readmissions were HE recurrence in 24 patients (21.4%), ascites in 15 patients (13.4%), infections in four patients (3.6%), and portal hypertensive bleeding in three patients (2.7%) (Table 3). Figure 2A shows the probability of hospital readmission for further decompensation events in the HE group. Six- and twelve-month rates of hospital readmission were 43.4% and 51.5%, respectively. Six- and twelve-month rates of HE recurrence were 25.1% and 27.7%, respectively. Six- and twelve-month rates of ascites recurrence were 11.6% and 14.3%, respectively. Six- and twelve-month rates of infections were 2.8% and 5.7%, respectively. Six- and twelve-month rates of bleeding were 3.8%. No significant risk factors for HE recurrence were found by univariate analysis. Probabilities of HE recurrence according to the previous history of HE are shown in Supplementary Figure S5.

In the no HE group, 68 patients (23.4%) had at least one hospital readmission, while 188 patients (73.4%) had no hospital readmission. The reasons for hospital readmissions were ascites in 27 patients (10.5%), HE in 27 patients (10.5%), portal hypertensive bleeding in 15 patients (5.9%), and infections in 13 patients (5.1%) (Table 3). Figure 2B shows the probability of hospital readmission for further decompensation events in the no HE group. Six- and twelve-month rates of hospital readmission were 26.2% and 70%, respectively. Six- and twelve-month rates of portal hypertensive bleeding were 4.4% and 37.8%, respectively. Six- and twelve-month rates of ascites were 8.9% and 12.2%, respectively. Six- and twelve-month rates of HE were 8.5% and 11.8%, respectively. Six- and twelve-month rates of infections were 4.5% and 7.1%, respectively. More details on the number and reasons for hospital readmission in the two groups are presented in Supplementary Table S3.

## Discussion

In this prospective multicenter study including decompensated cirrhotic patients hospitalized for HE age, HE, ascites, and hyponatremia were independent risk factors for death by multivariable competing risks analysis. HE increased not only the risk of death but also represented the first cause of hospital readmission compared with other decompensating events.

Up to now, concerns remain about which patients with HE should be referred to a transplant center because HE is not in itself an indication of OLT unless it is associated with advanced liver failure as assessed by the MELD score. However, it has been recently shown that the MELD score suffers from poor accuracy and calibration for predicting short-term mortality (15), and that the severity of liver disease may be underestimated by MELD alone in patients with HE (16). To the best of our knowledge, we demonstrated for the first time that not only patients with recurrent or persistent HE but also with a single episode of HE had a significantly worse survival by multivariable competing risks analysis; thus, one of the main implications for clinical practice of our findings is that patients with a single HE episode requiring hospitalization should be promptly considered for OLT. These findings could potentially contribute to future changes in clinical guidelines.

As expected, ascites was confirmed as an independent risk factor for death both in the whole cohort and in the HE group. Notably, the negative impact of HE on the survival of patients with cirrhosis was independent of other complications of advanced chronic liver disease, including ascites and hyponatremia. While the development of ascites mainly depends on the severity of portal hypertension, HE is related not only to portal hypertension, which leads to the opening of spontaneous porto-systemic shunts, but also reflects other pathophysiological mechanisms, such as impaired nutritional status, sarcopenia, and frailty (17, 18). It is not surprising that in our study, patients with HE had not only a significantly higher prevalence of spontaneous porto-systemic shunts assessed by CT but also significantly lower albumin levels, lower BMI, and higher MELD score compared with patients with decompensated cirrhosis without HE. The incorporation of novel variables related to nutritional status and chronic inflammation could potentially improve the allocation systems for OLT (19, 20).

Body mass index (BMI) was significantly lower in the HE group compared with the no HE group, and it was independently associated with worse survival in the HE group. This last finding suggests the relevance of nutritional status as a determinant of clinical outcomes in patients with cirrhosis. Decompensated cirrhosis represents a hyper-metabolic state, resulting in accelerated protein catabolism and higher energy consumption (21). Although BMI is not a widely accepted marker of nutritional status, particularly in patients with ascites, previous studies have shown that lower BMI is related to sarcopenia and decreased muscle mass in patients with cirrhosis (22), and that sarcopenia is a significant predictor of death independently from the degree of liver dysfunction (23). The role of BMI appears particularly relevant since our cohort had a relatively low prevalence of metabolic cirrhosis. Future research should investigate the role of lumbar muscle cross-sectional area measured by CT or other measures of muscle strength as surrogate biomarkers of sarcopenia in predicting the risk of death in patients with cirrhosis and HE. Moreover, further studies are needed to assess if combining standard medical treatment with nutritional interventions, physical exercise, and hormone-substituting therapies aiming to improve sarcopenia and muscle strength may be able to reduce the risk of death and HE recurrence.

The prospective design of our study allowed us to accurately estimate the risk of hospital readmission for HE recurrence. It is important to underline that the 12-month risk of HE recurrence was high, and that HE recurrence was the first leading cause of hospital readmission compared with other further decompensation events, including ascites, infections, and portal hypertensive bleeding. These findings underline the significant burden of HE on healthcare systems, in terms of repeated hospital admissions and related direct and indirect costs (8, 9, 24). Particularly, the results of our prospective study confirmed those of previously published retrospective studies, showing that HE is a strong predictor of early hospital readmission in patients with cirrhosis (25–27). Notably, the risk of HE recurrence was high, regardless of the characteristics of previous HE episodes (episodic or recurrent), suggesting that secondary prophylaxis should be recommended in clinical practice after the first HE episode. The prospective design of our study and the accurate assessment of received pharmacological treatments and outcomes during the follow-up can be useful in order to design future pharmacoeconomic studies. These latter are needed to evaluate the cost-effectiveness of both secondary prophylaxis and treatment strategies, including rifaximin, lactulose, or branched-chain amino acids.

Our study was also able to provide updated data on the prevalence of precipitating events. Approximately 80% of HE patients showed a precipitating factor, the most prevalent being constipation, infections, diuretic overuse, and gastrointestinal bleeding. These results could be useful in order to plan effective primary prophylaxis strategies. Infections are now considered complications of decompensated cirrhosis (28), and they could accelerate the course of the disease, precipitating acute-on-chronic liver failure (29). However, effective strategies to prevent infections in decompensated cirrhosis are lacking. Although long-term therapy with albumin has showed to significantly reduce the risk of infections and grade III-IV HE improving survival in a randomized controlled trial (RCT) including outpatients with cirrhosis and uncomplicated ascites (30), this beneficial effect was not confirmed in hospitalized patients with more advanced disease treated with a short course of albumin (31). An RCT including Child–Pugh C patients showed that long-term norfloxacin significantly decreased the incidence of any gram-negative bacterial infections, but it failed to improve 6-month mortality (32). The use of rifaximin for the prevention of infections is not accepted since evidence from clinical trials is still weak (33). Fecal microbiota transplant (FMT) has shown to be safe and potentially efficacious for the treatment of HE (34), and its potential role in preventing infections is currently under evaluation (35). About gastrointestinal bleeding as precipitating factor of HE, the last Baveno VII recommendations suggested the rapid removal of blood from the gastrointestinal tract by using lactulose oral or enemas in order to prevent HE (33). Although it has been shown that PPI are associated with overt HE and increased mortality (36), it is noteworthy that a high prevalence of PPI use was observed among decompensated cirrhotic patients, suggesting that more stringent criteria for PPI prescription should be used in this setting.

Our study suffers from some limitations. First, the enrollment of patients by 25 referral centers may have affected our results, since discrepancies in the HE grading between observers, treatment and prophylaxis strategies, and policy of OLT allocation may differ among centers. However, according to recently published EASL guidelines, our study used a standardized flow-chart for differential diagnosis, and only patients with the diagnosis of at least grade 2 overt HE as a reason for hospitalization were included. Second, treatments and prophylaxis strategies for HE were highly heterogeneous among centers, and no firm conclusions regarding the efficacy and safety of treatments, including lactulose, rifaximin, albumin, and beta-blockers, could be derived. However, the results of our multivariable competing risks model were confirmed after adjusting for the center effect as a covariate. Third, our sample size was relatively small. Fourth, our study only included hospitalized patients with HE; therefore, our findings could not be generalized to outpatients with HE. Fifth, even if multicenter, our study included patients who were enrolled only in Italian centers; therefore our results need to be validated in other settings, with different prevalences of etiologies of liver disease or metabolic co-factors affecting survival. Finally, although all patients in the HE group were consecutively included during the enrollment period, the same did not apply to the no HE group, potentially introducing a selection bias in the control group.

In conclusion, in decompensated cirrhotic patients hospitalized for HE as an inception point, HE is an independent risk factor for mortality, and it is the most common cause of hospital readmission compared with other decompensation events. In patients with HE, ascites and BMI were independent risk factors for death, suggesting that improvement of sarcopenia represents an urgent unsolved medical need in this setting.

## Data availability statement

The raw data supporting the conclusions of this article will be made available by the authors, without undue reservation.

## Ethics statement

The studies involving human participants were reviewed and approved by Institutional Review Board (IRB) of the Fondazione Agostino Gemelli Hospital, Rome, Italy. The patients/participants provided their written informed consent to participate in this study.

## Author contributions

OR, CCe, VC, MMe, PC, SM, VM, MMi, GSa, GR, AB, PB, RS, MPe, FS, EV, AC, SF, MPi, MB, FA, GSo, MR, FM, SL, AF, AP, GD, SN, LR, AG, and CCa were responsible for the project and writing of the manuscript. All authors have seen and approved the final version of the manuscript.
