# Supplementary material for: Hepatic encephalopathy increases the risk for mortality and hospital readmission in decompensated cirrhotic patients: a prospective multicenter study
Source: Front Med (Lausanne). 2023 May 25;10:1184860. doi: 10.3389/fmed.2023.1184860 (PMC10248517; doi:10.3389/fmed.2023.1184860)
Supplement: Supplementary file 1 [file Data_Sheet_1.docx]

**Hepatic encephalopathy increases the risk for mortality and hospital readmission in decompensated cirrhotic patients: a prospective multicentre study**

**Oliviero Riggio*, Ciro Celsa*, Vincenza Calvaruso, Manuela Merli, Paolo Caraceni, Sara Montagnese, Vincenzina Mora, Martina Milana, Giorgio Maria Saracco, Giovanni Raimondo, Antonio Benedetti, Patrizia Burra, Rodolfo Sacco, Marcello Persico, Filippo Schepis, Erica Villa, Antonio Colecchia, Stefano Fagiuoli, Mario Pirisi, Michele Barone, Francesco Azzaroli, Giorgio Soardo, Maurizio Russello, Filomena Morisco, Sara Labanca, Anna Fracanzani, Antonello Pietrangelo, Gabriele Di Maria, Silvia Nardelli, Lorenzo Ridola, Antonio Gasbarrini**, Calogero Cammà****

***these authors shared first authorship**

**** these authors shared senior authorship**

**List of supplementary Materials**

Page 2. Algorithm to exclude causes of altered mental status other than hepatic encephalopathy

Page 5. Definition of precipitating events

Page 7. Sample size calculation. Supplementary Table S1.

Page 9. Supplementary Table S2.

Page 10. Supplementary Table S3.

Page 11. Supplementary Figure S1.

Page 12. Supplementary Figure S2.

Page 13. Supplementary Figure S3.

Page 14. Supplementary Figure S4.

Page 15. Supplementary Figure S5.

**Algorithm to exclude causes of altered mental status other than hepatic encephalopathy**

1. **Exclusion of hypercapnic encephalopathy.**

- Respiratory failure? History of respiratory or neuromuscular disease?

If no, go to step 2.

If yes, assess partial pressure of carbon dioxide (pCO2)

- pCO2>60 mmHg?

If no, go to step 2.

If yes: **patient excluded** (hypercapnic encephalopathy)

1. **Exclusion of** **hypoglycemic encephalopathy**

- Blood glucose levels (BGL) < 50 mg/dL?

If no: go to step 3.

If yes: administer glucose and re-evaluate consciousness after normalization of BGL

- Neurological manifestations improved after normalization of BGL?

If no: go to step 3.

If yes: **patient excluded** (hypoglicemic encephalopathy)

1. **Exclusion of hyperosmolar encephalopathy**

- Serum osmolality > 320 mOsm/kg? (calculated as 2 [Na+ (mEq/L)]+glucose (mg/dL)/18)

If no: go to step 4.

If yes: **patient excluded** (hyperosmolar encephalopathy)

1. **Exclusion of organic brain disease**

- Clinical presentation with one of the following: hemiparesis, hemianopsia, aphasia, severe headache, recent traumatic brain injury?

If no: go to step 5.

If yes: perform brain computed tomography (CT) or magnetic resonance imaging (MRI).

- Radiological evidence of organic brain injury?

If no: go to step 5.

If yes: **patient** **excluded.**

- High suspicion of subarachnoid haemorrhage despite negative imaging?

If no: go to step 5.

If yes: perform lumbar puncture.

1. **Exclusion of meningo-encephalitis**

- Severe headache or meningeal syndrome?

If no: go to step 6.

If yes: lumbar puncture (after exclusion of increased intracranial pressure by CT or MRI). If abnormal cerebrospinal fluid analysis: **patient** **excluded.**

1. **Exclusion of nonconvlusive status epilepticus**

- History of seizures or myoclonus?

If no: go to step 7.

If yes: perform electroencephalogram (EEG). Decision to enrol or not the patient according to EEG results.

1. **Exclusion of alcohol abuse**

- History or signs of recent alcohol abuse?

If no: go to step 8.

If yes: measure blood alcohol levels. If high, **patient excluded.**

1. **Exclusion of alcohol withdrawal syndrome**

- Regular alcohol consumption of at least 80 gr/day recently discontinued and psychomotor agitation with visual hallucinations?

If no: go to step 9.

If yes: **patient excluded.**

1. **Exclusion of benzodiazepine abuse**

- History of benzodiazepine use?

If no: go to step 10.

If yes: measure serum and urine benzodiazepines and administer flumazenil. If significant clinical improvement within first minutes: **patient excluded.**

1. **Exclusion of drug abuse**

- History of consumption of drugs that can decrease consciousness?

If no: go to step 11.

If yes: if possible, measure serum or urine levels. If abnormal values or consumption of unmeasurable drug: **patient excluded**

1. **Exclusion of Wernicke encephalopathy**

- Clinical presentation with ophthalmoplegia, ataxia and loss of short-term memory?

If no: go to step 12.

If yes: measure blood thiamine concentration or perform brain MRI. If abnormal: **patient excluded.**

1. **Exclusion of alternative causes**

- Other etiology of encephalopathy different from hepatic encephalopathy are suspected?

If no: **patient can be enrolled**

If yeas: **patient excluded.**

**Definition of precipitating events**

1. **Gastrointestinal bleeding**

Definition: overt bleeding from gastrointestinal tract

Mandatory investigations:

- assess the presence of hematemesis, melena, and hematochezia
- assess anemia or iron deficiency with complete blood count, serum iron, ferritin and transferrin.
- upper or lower endoscopy

1. **Infections**

Definitions:

- Pneumonia: presence on chest X-ray of new pulmonary infiltrate, consolidation, or cavitation associated with at least one of the following signs/symptoms (onset of purulent sputum or changes in its appearance, onset of cough, pleuritic chest pain, pulmonary crepitations, bronchial hums or noises, worsening of gas exchange) and/or culture test positivity on pleural fluid or samples obtained by trans-tracheal, aspirate, or broncho-alveolar lavage (BAL).
- Urinary tract infection: at least one of the following symptoms (urinary urgency, pollakiuria, dysuria, hematuria, or suprapubic pain) and a positive urine culture or at least 2 of the following symptoms (urinary urgency, pollakiuria, dysuria, hematuria, or suprapubic pain) and at least 10 leukocytes/mL on urine physical-chemical examination
- spontaneous bacterial peritonitis (SBP): presence of polymorph nuclear leukocytes ≥ 250 cells/mm3 in the ascitic fluid
- bacteremia: positive blood cultures (at least 2 for skin contaminants) in the absence of a known source of infection
- other infections: diagnosis according to Centers for Disease Control and Prevention criteria

Mandatory investigations:

- blood culture, urine culture, and chest X-ray.
- paracentesis with cell counts
- stool cultures in patients with diarrhea (>4 discharges per day)
- sputum culture examination in patients with productive cough.

1. **Constipation**

Definition: no bowel movements for at least 24 hours or demonstration of significant faecal retention.

Mandatory investigations:

- Assess stool frequency
- Rectal examination

1. **Diuretics**

Definition: temporal relationship between hepatic encephalopathy and diuretics use.

Mandatory investigations:

- Assess use and dosage of diuretics
- Measure blood acid-base balance, serum creatinine, uraemia, sodium and potassium levels.

1. **Dehydration**

Definition: clinical and biochemical signs of dehydration (dry mucous membranes, increased haematocrit, creatinine, uraemia or sodium levels)

Mandatory investigations:

- Assess excessive sweating, vomiting, or diarrhoea
- Measure blood acid-base balance, serum creatinine, uraemia, sodium and potassium levels.

**Methods**

**Statistical analysis**

Sample size calculation: To estimate the number of patients with cirrhosis hospitalized for hepatic encephalopathy compared to patients hospitalized for other decompensation events of cirrhosis, hypothesizing a prevalence of 30% (with a precision of 5%), with an alpha error of 0.05, 325 patients will be needed. The applied formula is:

n = (Z 1- α)^2^ (P (1-P)/D^2^)

were Z .95 = 1.96; P = 0.30; D =0.05

Considering an attrition rate at the end of follow-up of 12% (39 patients), the study should enrol 364 patients.

**Supplementary Table S1. Risk factors for death and orthotopic liver transplant by univariate competing risks analysis.**

|  | **Death** | | | **Liver transplant** | | |
| --- | --- | --- | --- | --- | --- | --- |
|  | **HR** | **95% CI** | **p-value** | **HR** | **95% CI** | **p-value** |
| Age (years) | 1.03 | 1.00-1.05 | 0.02 | 0.96 | 0.95-0.98 | <0.001 |
| Male sex (%) | 1.33 | 0.83-2.13 | 0.23 | 1.31 | 0.73-2.34 | 0.37 |
| Etiology of liver disease (%)  Viral  Alcohol  Autoimmune  Biliary  Metabolic  Others | 1.17  0.71  1.13  1.15  1.05  1.81 | 0.74 – 1.85  0.48 – 1.07  0.33 – 3.87  0.45 – 2.96  0.59 – 1.89  0.91 – 3.6 | 0.5  0.10  0.84  0.77  0.87  0.10 | 1.08  0.89  1.53  2.03  0.73  0.97 | 0.62 – 1.89  0.55 – 1.44  0.52 – 4.54  0.7 – 5.93  0.32 – 1.7  0.37 – 2.56 | 0.77  0.64  0.44  0.19  0.47  0.95 |
| Hospitalization for HE (vs hospitalization for other reasons) | 1.77 | 1.16-2.68 | 0.01 | 0.84 | 0.48-1.47 | 0.54 |
| BMI (kg/m2) | 0.99 | 0.95-1.03 | 0.51 | 0.99 | 0.94-1.05 | 0.79 |
| Haemoglobin (g/dL) | 1.01 | 0.92-1.11 | 0.81 | 0.97 | 0.85-1.10 | 0.60 |
| Haematocrit (%) | 0.99 | 0.96-1.03 | 0.75 | 0.99 | 0.95-1.04 | 0.81 |
| WBC (mmc) | 1.00 | 0.99-1.01 | 0.55 | 1.00 | 0.99-1.01 | 0.25 |
| PLT (10^9^/L) | 1.00 | 0.99-1.01 | 0.81 | 1.00 | 0.99-1.01 | 0.68 |
| ALT (U/mL) | 1.00 | 0.99-1.01 | 0.23 | 1.00 | 0.99-1.01 | 0.30 |
| GGT (U/mL) | 1.01 | 1.00-1.02 | 0.04 | 1.00 | 0.99-1.01 | 0.39 |
| Total bilirubin (mg/dL) | 0.99 | 0.96-1.04 | 0.80 | 0.98 | 0.94-1.02 | 0.37 |
| Creatinine (mg/dL) | 1.08 | 0.83-1.39 | 0.57 | 1.15 | 0.75-1.78 | 0.52 |
| Albumin (g/dL) | 0.93 | 0.65-1.34 | 0.70 | 0.90 | 0.62-1.30 | 0.57 |
| INR | 0.90 | 0.60-1.35 | 0.61 | 0.93 | 0.55-1.58 | 0.79 |
| Sodium (mEq/L) | 0.94 | 0.90-0.99 | 0.01 | 0.99 | 0.95-1.04 | 0.81 |
| Venous Blood Ammonia (µmol/L) | 1.00 | 0.99-1.01 | 0.60 | 1.00 | 0.99-1.01 | 0.48 |
| Ascites (present vs absent) | 2.32 | 1.42 - 3.79 | <0.001 | 0.907 | 0.548 – 1.5 | 0.71 |
| Child-Pugh class (class C versus classes A and B) | 2.15 | 1.44-3.22 | <0.001 | 1.33 | 0.81-2.17 | 0.26 |
| MELD score | 1.01 | 0.98-1.04 | 0.67 | 0.99 | 0.96-1.03 | 0.61 |
| MELD-Na score | 1.02 | 0.99-1.04 | 0.26 | 1.00 | 0.93-1.03 | 0.76 |
| Gastroesophageal varices (present vs absent) | 0.915 | 0.54 – 1.56 | 0.74 | 1.23 | 0.61 – 2.49 | 0.56 |
| Portal thrombosis | 1.39 | 0.76-2.56 | 0.28 | 1.04 | 0.50-2.16 | 0.91 |
| Porto-systemic shunts | 1.11 | 0.66-1.88 | 0.69 | 0.97 | 0.54-1.73 | 0.92 |
| Hepatocellular carcinoma | 1.01 | 0.59-1.74 | 0.97 | 1.23 | 0.69-2.20 | 0.49 |
| Presence of TIPS | 0.90 | 0.30-2.71 | 0.85 | 1.62 | 0.58-4.56 | 0.36 |

HE, hepatic encephalopathy. BMI, body mass index. WBC, white blood cells. PLT, platelets. AST, alanine aminotransferases. GGT, gamma-glutamil transferase. INR, international normalized ratio. MELD, model for end-stage liver disease.

**Supplementary Table S2. Risk factors for mortality and liver transplant in the whole cohort of cirrhotic patients (n = 368) hospitalized for decompensating events by multivariable competing risks analysis, after adjustment for baseline covariates significantly different between patients admitted to hospital for HE (HE group) or for other decompensating events (no HE group).**

|  | **Death** | | | **Liver transplant** | | |
| --- | --- | --- | --- | --- | --- | --- |
|  | **HR** | **95% CI** | **p-value** | **HR** | **95% CI** | **p-value** |
| Age (years) | 1.03 | 1.01 - 1.05 | 0.031 | 0.96 | 0.94 - 0.99 | 0.001 |
| Male sex (versus female sex) | 1.26 | 0.75 - 2.09 | 0.379 | 1.34 | 0.73 - 2.48 | 0.342 |
| BMI (kg/m2) | 0.99 | 0.95 - 1.04 | 0.784 | 0.98 | 0.94 - 1.03 | 0.479 |
| HE (present vs absent) | 1.62 | 1.01 - 2.58 | 0.043 | 1.04 | 0.54 - 2 | 0.909 |
| Ascites (present vs absent) | 2.75 | 1.59 - 4.76 | <0.0001 | 1.09 | 0.6 - 1.95 | 0.784 |
| HCC (present vs absent) | 1.37 | 0.78 - 2.39 | 0.273 | 1.66 | 0.89 - 3.11 | 0.112 |
| TIPS (present vs absent) | 0.67 | 0.18 - 2.54 | 0.553 | 1.68 | 0.5 - 5.57 | 0.398 |
| Porto-systemic shunts (present vs absent) | 0.93 | 0.57 - 1.52 | 0.765 | 1.3 | 0.74 - 2.29 | 0.354 |
| Sodium (mEq/L) | 0.95 | 0.91 - 0.99 | 0.019 | 0.99 | 0.94 - 1.04 | 0.701 |
| Hemoglobin (g/dL) | 1.02 | 0.91 - 1.14 | 0.723 | 1.03 | 0.89 - 1.19 | 0.709 |
| GGT (U/mL) | 1 | 0.99 – 1.01 | 0.058 | 1 | 1 - 1 | 0.860 |
| Albumin (g/dL) | 1.02 | 0.69 - 1.49 | 0.936 | 0.94 | 0.59 - 1.51 | 0.811 |

Results are adjusted for centre effect.

HR, hazard ratio. 95%CI, 95% confidence interval. BMI, body mass index. HE, hepatic encephalopathy. HCC, hepatocellular carcinoma. TIPS, transjugual intrahepatic porto-systemic shunt. GGT, gamma-glutamil transferase.

**Supplementary Table S3. Reasons for hospital readmission in 112 hospitalized patients with cirrhosis and hepatic encephalopathy.**

|  | **HE group**  **(n=112)** | **No HE group (n=256)** |
| --- | --- | --- |
| Number of hospital readmission for hepatic encephalopathy  0  1  2  ≥3 | 88 (71.4)  18 (16.1)  4 (3.6)  2 (1.8) | 229 (89.5)  21 (8.2)  5 (1.9)  1 (0.4) |
| Number of hospital readmission for ascites  0  1  2  ≥3 | 97 (86.6)  11 (9.8)  2 (1.8)  2 (1.8) | 229 (89.5)  20 (7.8)  7 (2.7)  0 (0.0) |
| Number of hospital readmission for infections  0  1  2 | 108 (96.4)  2 (1.8)  2 (1.8) | 243 (94.9)  9 (3.5)  4 (1.6) |
| Number of hospital readmission for portal hypertensive bleeding  0  1  2  ≥3 | 109 (97.3)  2 (1.8)  1 (0.9)  0 (0.0) | 241 (94.1)  10 (3.9)  4 (1.6)  1 (0.4) |

HE, hepatic encephalopathy

**Supplementary Figure S1. Study flow chart**

**Supplementary Figure S2. Probability of death and orthotopic liver transplant in 368 patients hospitalized for decompensated cirrhosis.**

**
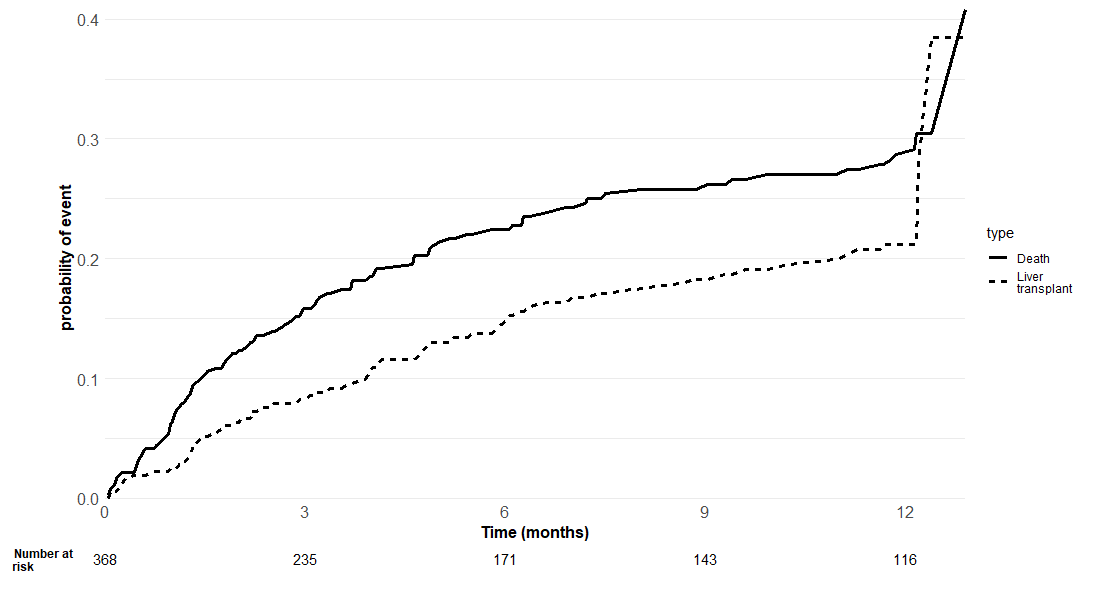
**

**Supplementary Figure S3. Probability of orthotopic liver transplant in 368 hospitalized patients with decompensated cirrhosis stratified according to the reason of hospitalization (hepatic encephalopathy versus no hepatic encephalopathy)**

**
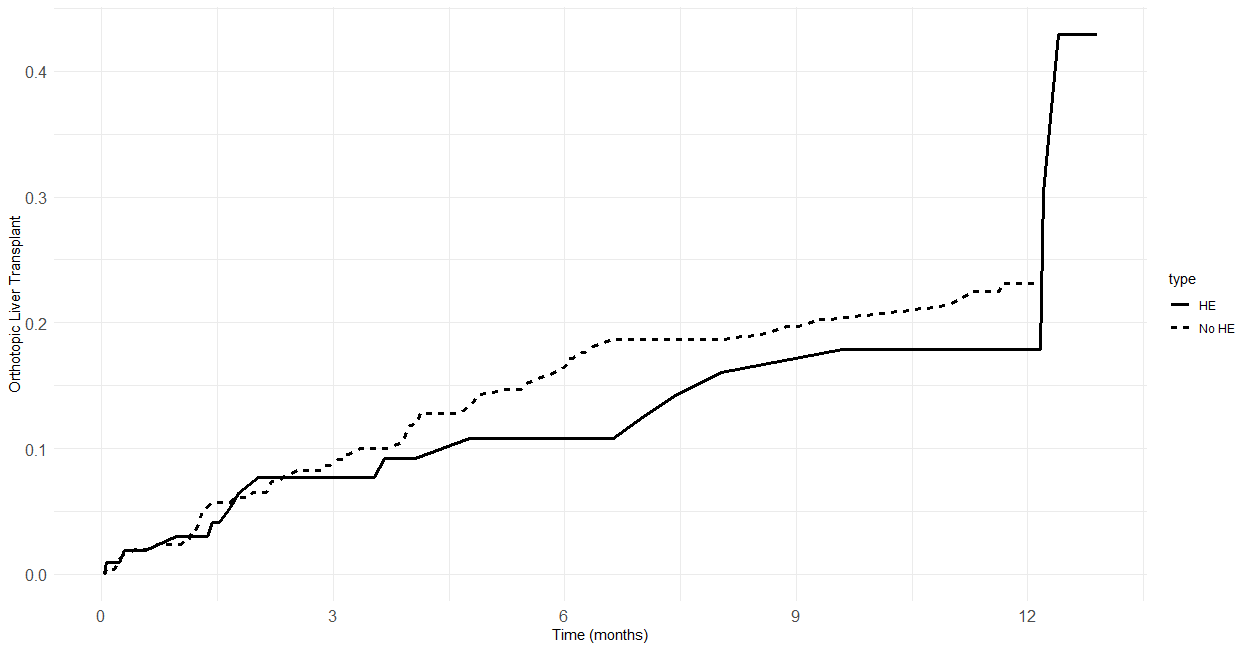
**

**Supplementary Figure S4. Predicted probabilities of death in 368 patients hospitalized for decompensated cirrhosis in four different patient profiles. A: Presence of ascites and hepatic encephalopathy. B: presence of ascites without hepatic encephalopathy. C: presence of hepatic encephalopathy without ascites. D: absence of ascites and hepatic encephalopathy.**


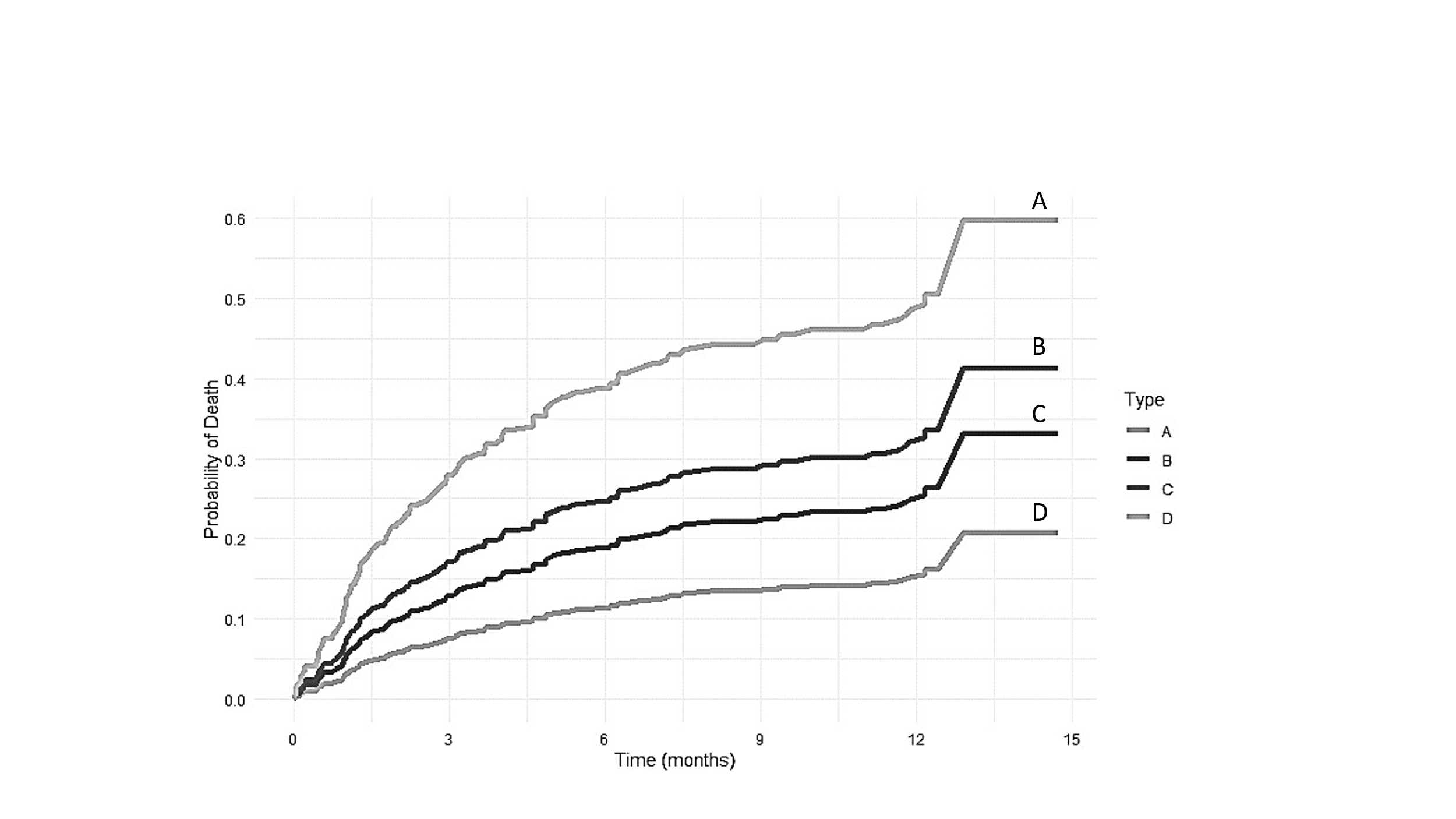


**Supplementary Figure S5. Probability of hepatic encephalopathy (HE) recurrence according to previous history of HE in HE group.**

**
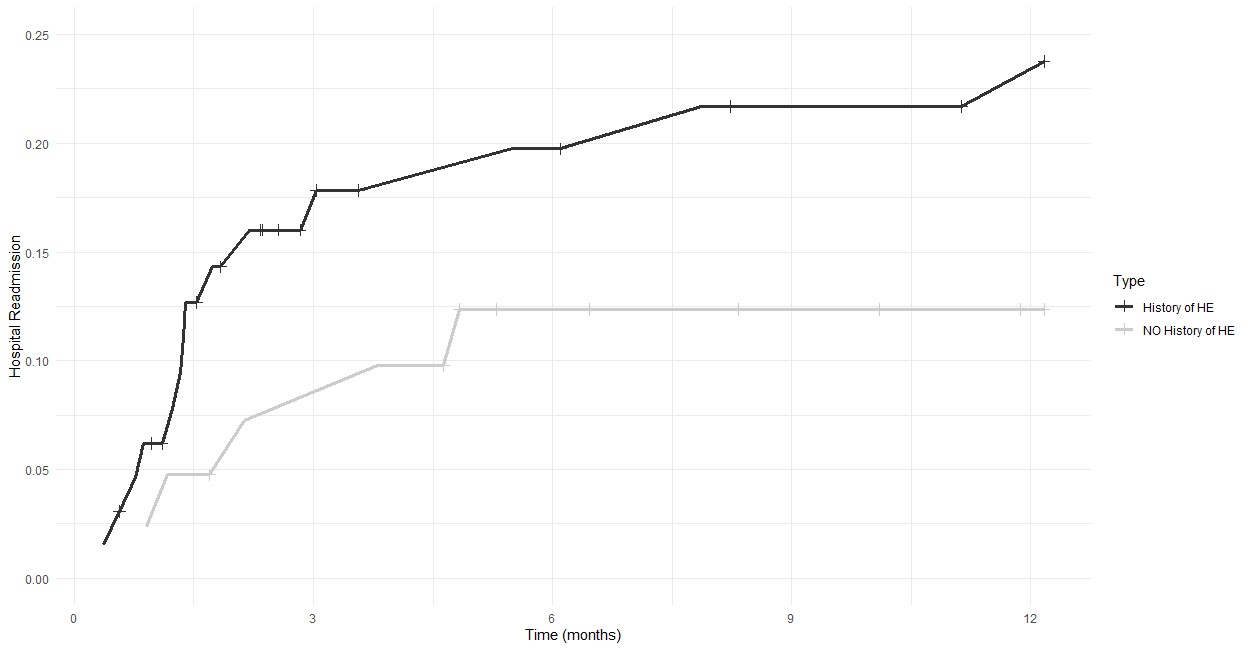
**
